# Well-digging in a community of forest-living wild East African chimpanzees (*Pan troglodytes schweinfurthii*)

**DOI:** 10.1007/s10329-022-00992-4

**Published:** 2022-06-06

**Authors:** Hella Péter, Klaus Zuberbühler, Catherine Hobaiter

**Affiliations:** 1 School of Psychology and Neuroscience, University of St Andrews, St Andrews, UK; 2 School of Anthropology and Conservation, University of Kent, Canterbury, UK; 3 Department of Comparative Cognition, University of Neuchâtel, Neuchâtel, Switzerland; 4 Budongo Conservation Field Station, PO Box 362, Masindi, Uganda

**Keywords:** Culture, Social transmission, *Pan troglodytes*, Water access, Hydration

## Abstract

Access to resources shapes species’ physiology and behaviour. Water is not typically considered a limiting resource for rainforest-living chimpanzees; however, several savannah and savannah-woodland communities show behavioural adaptations to limited water. Here, we provide a first report of habitual well-digging in a rainforest-living group of East African chimpanzees (*Pan troglodytes schweinfurthii*) and suggest that it may have been imported into the community’s behavioural repertoire by an immigrant female. We describe the presence and frequency of well-digging and related behaviour, and suggest that its subsequent spread in the group may have involved some degree of social learning. We highlight that subsurface water is a concealed resource, and that the limited spread of well-digging in the group may highlight the cognitive, rather than physical, challenges it presents in a rainforest environment.

**Supplementary Information:**

The online version contains supplementary material available at 10.1007/s10329-022-00992-4.

## Introduction

Access to resources shapes species’ physiology and behaviour across taxa, e.g. foraging-related differences in bill shape in parrots (Homberger 2003; Froggatt and Gill 2016), or the distinctive probe-like morphology of the middle finger of the aye-aye (*Daubentonia madagascariensis*) (Sterling and McCreless 2006). Occasionally, resources are concealed or difficult to access in ways which present a particular cognitive challenge that is addressed through behavioural adaptations. Good examples of this include the complex manual neutralisation of plant defences in mountain gorillas (*Gorilla gorilla beringei*) (Byrne and Byrne 1993), and the extraction of nuts from their hard outer shell, as seen in corvids [*Corvus brachyrhynchos* (Cristol and Switzer 1999); *Corvus moneduloides* (Hunt et al. 2002)] and chimpanzees (*Pan troglodytes*) (Savage and Wyman 1843/1844; Boesch and Boesch 1983), which involves the planning of complex manipulations to extract items that are not visibly present.

Water, a resource of universal relevance, is rarely considered a concealed resource; it is usually directly accessible from surfaces, cavities, or other types of containers. However, water is also present beneath the surface, where access is only possible through the creation of wells*.* Some species have been documented to regularly exploit concealed water. Reports include those on African elephants (*Loxodonta africana*) (Epaphras et al. 2007; Ramey et al. 2013; Stommel et al. 2016), warthogs (*Phacochoerus africanus*) (Stommel et al. 2016) and various equids, such as feral horses (*Equus ferus caballus*) and donkeys (*Equus ferus caballus*) (Lundgren et al. 2021), khulan (*Equus hemionus kulan*) (Payne et al. 2020), mountain zebras (*Equus zebra*) (Klingel 1968) and plains zebra (*Equus quagga*) (Epaphras et al. 2007; Stommel et al. 2016). In all of these cases, the animals live in environments with extremely low and seasonal rainfall, necessitating adaptations to access water. Most non-human primate species live in habitats with low levels of aridity (Stone et al. 2013; Wessling et al. 2020) where water is not usually limited, and is often an abundant resource. Nevertheless, we are aware of reports of well-digging in four primate species: hamadryas baboons (*Papio hamadryas*) (Biquand et al. 1992), which are reported to dig wells of up to 20 m in depth (Biquand et al. 1992); chacma baboons (*Papio ursinus*), which occasionally excavate their own wells (Hamilton et al. 1985), but more often re-excavate existing seeps (Brain 1990) or deepen wells dug by gemsbok (*Oryx gazella*) (Hamilton et al. 1978, 1985) or jackals (*Canis mesomelas*) (Hamilton et al. 1978); yellow baboons (*Papio cynocephalus*) (Stommel et al. 2016); and savannah-woodland- and savannah-dwelling chimpanzee communities that have been reported to dig wells at field sites in Tanzania, Uganda, and Senegal (Nishida et al. 1999, 2010; Hunt 2000, 2020; McGrew et al. 2003; Hunt and McGrew 2002; Galat et al. 2008; Galat-Luong et al. 2009).

Most chimpanzee communities live in rainforests, an environment in which water is rarely a limiting resource. However, even in rainforests, periods of water shortage can occur, either because of seasonal variation in rainfall [e.g. in the Taï forest, Côte d’Ivoire (Wessling et al. 2018)] or because of specific hydrologic situations, such as in Tongo, Democratic Republic of Congo, where volcanic soil absorbs water rapidly from the surface (Lanjouw 2002). In communities that live in arid and open savannah and savannah-woodland habitats with limited water availability [e.g. Fongoli and Mt Assirik, Senegal (Pruetz et al. 2002; McGrew et al. 1981); Toro-Semliki, Uganda (Hunt and McGrew 2002); Issa, Tanzania (Hernandez-Aguilar 2006)], a number of behavioural adaptations to deal with dehydration and heat stress have been observed, including increased nocturnal activity (Pruetz 2018), the use of caves and water pools for thermoregulation (Pruetz 2007), and the consumption of underground tubers primarily for water, rather than as food (Lanjouw 2002).

The digging of small wells to access water in dry environments has been documented in three chimpanzee communities to date. In Mahale, Tanzania, chimpanzees have occasionally been observed to dig in a dried-out creek bed to reach the water table, both manually and by using a stick tool (Nishida et al. 1999). Manual well-digging is habitually observed in two arid long-term chimpanzee study sites at Mt Assirik, Senegal (McGrew et al. 2003; Galat et al. 2008; Galat-Luong et al. 2009) and Toro-Semliki, Uganda (Hunt and McGrew 2002; McGrew et al. 2007).

The majority of well-digging at these sites occurred during periods of lower-than-average rainfall; however, wells were not only found in dried-out riverbeds, but also next to free-flowing surface water, suggesting additional benefits, such as the filtering out of debris or contaminants, as compared to drinking directly from the open water. To date, only a few species have been suggested to dig wells in order to improve the potability of water. Stagnant, non-flowing water can harbour an increased load of pathogens (Felföldi et al. 2010; Lambrecht et al. 2016) and parasites (Southgate 2009), and species such as African elephants (Ndlovu et al. 2018) and red-fronted lemurs (*Eulemur rufifrons*) (Amoroso et al. 2019) actively avoid such contaminated water sources. A preliminary report suggested that bacterial loads of water in wells dug by chimpanzees in Senegal may have been up to ten times lower than that of nearby stagnant water (Galat et al. 2008). In Semliki, Uganda, water from wells dug in sand appeared to have lower alkalinity than that of a nearby free-flowing river (Hunt 2020). In African elephants, the use of wells to ‘filter’ water increased as bacterial loads increased (Ramey et al. 2013; Stommel et al. 2016) and water was cleaner and cooler than in other natural water holes (Epaphras et al. 2007). Hamadryas baboons appear to similarly prefer apparently clear filtered well water over stagnant sources (Kummer 1971). As a result, well-digging may represent an adaptation, not only for accessing water when other sources such as creeks or rivers are dry, but also as a means of improving water quality.

Behaviour, e.g. well-digging, may be an adaptation to the physical environment acquired through genetic endowment or individual learning. However, some behaviours in chimpanzees are transmitted through social learning, which is a key criterion for a behaviour to qualify as cultural (Laland and Hoppitt 2003). Examples of animal cultural behaviours are from studies on humpback whales (*Megaptera novaeangliae*) (Owen et al. 2019), chaffinches (*Fringilla coelebs*) (Riebel et al. 2015), spider monkeys (*Ateles geoffroyi*) (Santorelli et al. 2011)) and, most importantly, in terms of the diversity and volume of evidence, from chimpanzees (Whiten et al. 1999; Kalan et al. 2020). Although acquisition through social learning is very plausible for most group-specific behaviours, direct evidence for social learning is difficult to obtain in wild populations. Rare exceptions of this are the innovation and subsequent spread of the use of moss as a sponge material in the Sonso community of Budongo Forest (Hobaiter et al. 2014; Lamon et al. 2017), and the spread of ant fishing in the Kasekela community in Gombe (O’Malley et al. 2012). When direct observations are lacking, claims of cultural behaviours are normally based on the exclusion method, i.e. where explanations based on genetic or ecological causes for group differences are excluded because they are not supported or are less plausible than explanations based on social learning (Krützen et al. 2011; Lycett et al. 2010; Whiten et al. 1999; Kalan et al. 2020; but see Langergraber et al. 2010).

Potential sources of new cultural behaviours are innovations or imports by immigrant individuals. In chimpanzees, females usually disperse from their natal communities as subadults (Nishida et al. 2003), an age by which they are already competent tool users (Inoue-Nakamura and Matsuzawa 1997; Musgrave et al. 2020) and are thus likely candidates for transferring a tool-related behaviour between communities. In the Kasekela community, Tanzania, a new behaviour, ant fishing, was first observed in immigrant females who arrived from a neighbouring ant-fishing community, before it spread to resident individuals, although this only occurred in immature chimpanzees born after the females’ immigration (O’Malley et al. 2012). In the Bossou community, Guinea, a nut-cracking experiment with stone hammers was conducted with a new species of nuts. Remarkably, one of the adult females in the group showed immediate proficiency, suggesting that she learned the behaviour in her natal community (Biro et al. 2003). In the following years, other, immature members of the community also acquired the behaviour, which was widespread by the time the provisioning of the nuts ended (Biro et al. 2011). Primate archaeological evidence has also been used to provide support for the hypothesis that behaviour–here nut-cracking hammer selection—was transmitted between communities by migrating females (Luncz et al. 2015).

These examples present a conundrum. Although female migration provides regular opportunities for cultural behaviours to spread between communities, chimpanzee communities typically show stable long-term group differences in cultural behaviours (Nakamura and Ueahara 2004; Boesch et al. 2020). One possible explanation for this is conformity: individuals prefer the behavioural variants used by the majority, rather than the most efficient one, which prevents a new behaviour from spreading (Luncz and Boesch 2014; Whiten et al. 2005; Gruber et al. 2009, 2011; Grund et al. 2019). Another possible explanation is that an imported behaviour may not be subject to social learning. Although captive chimpanzees prefer to observe knowledgeable individuals, older and more dominant individuals are observed more often (Horner et al. 2010), which may disadvantage young low-ranking immigrant females as demonstrators of new behaviour (Biro et al. 2003). However, in other domains, immigrant females have been the source of social learning and subsequent group spread. During the habituation phase of the Waibira community, Uganda, it was observed that the immigration of two habituated females from the neighbouring Sonso community had an accelerating effect on habituation (Samuni et al. 2014). Nevertheless, even if novel behaviours are occasionally innovated in wild chimpanzees and brought to other communities by young females, subsequent spread appears to occur only rarely.

Here, we describe the appearance and subsequent spread of well-digging behaviour in a previously apparently naïve community of wild East African chimpanzees (*Pan troglodytes schweinfurthii*). Subadult female ONY immigrated to the Waibira community of the Budongo Forest, Uganda, in 2014. Shortly after her arrival, she was repeatedly seen digging wells in a water hole used by the community during the dry season. She dug small holes with her hand in the sandy-gravel substrate of the water hole, waited for water to filter through (0–13 s), then drank it. During these observations both mature and immature individuals were seen observing ONY digging with apparent interest (peering). Once ONY had finished drinking, the other individuals used her wells for both direct drinking and sponging up of water with tools made of leaves, moss, or a combination of both materials. Here we describe ONY’s well-digging behaviour and the same behaviour subsequently seen in other individuals of the community.

## Methods

**Fig. 1a–c:**
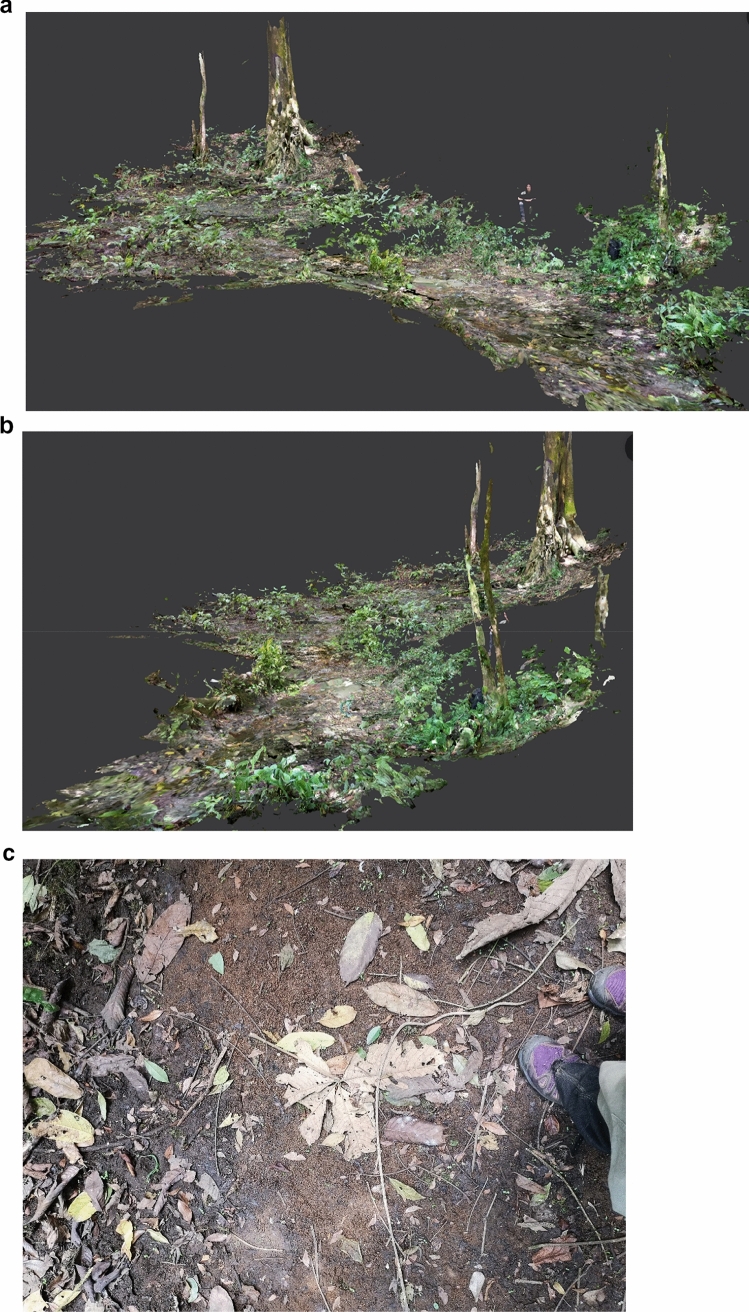
Light detection and ranging scan of the water hole area from two perspectives. **a** Overview of the water hole; camera trap 1 location is the small tree on the right. Camera trap 2 locations varied around the area of water below the base of the large tree, centre-left. **b** Perspective along the seasonal riverbed, camera trap 1 location is the tree in the foreground. **c** Photograph showing the two soil types found at the water hole in a dried-out section of the seasonal riverbed; the reddish sandy/gravel substrate in the centre is surrounded by the darker soil/loamy mud

The Waibira community lives in the Budongo Central Forest Reserve, Uganda. The community numbers ~ 120 individuals and is surrounded by an estimated four other communities. Habituation started in 2011 (Samuni et al. 2014) and staff and researchers from the Budongo Conservation Field Station follow individuals on a daily basis between 6 a.m. and 6 p.m. While they are a rainforest-living community in an area with high annual rainfall [1600 mm/year (Reynolds 2005)], there are no permanent rivers within the Waibira territory. During the main annual dry season (December-March) their primary drinking water access is restricted to a pool, the remaining section of a seasonal creek that runs through the centre of their territory. The pool consists of a roughly 10 × 10 m round area with shallow puddles mainly in sticky soil/loam-based mud and smaller areas of sandy-gravel substrate where the main creek bed runs during wet seasons, and a 6-m-long straight section to one side, which is the deepest part of the original creek (Fig. 1). Water flow is present through the rainy season, stops early during the dry season and does not resume until the end of it, meaning that the water is typically stagnant for 3 months, at the end of which it is very muddy and appears foul (i.e. dirty, with a filmy surface). Two similar pools are found in peripheral areas of the Waibira territory, but they overlap with the territories of neighbouring communities, making them more dangerous to access due to the risk of potentially lethal intercommunity encounters (Williams et al. 2008).

### Data collection

The central water hole has been monitored with Bushnell No Glow camera traps since January 2013 for every December-March dry season (2013–2017, single trap; 2017-present two traps). Camera traps were placed once rainfall started to decline (typically late November—early December) and before the chimpanzees started to visit the water hole. Camera traps were removed once the regular rains resumed and no chimpanzees had visited the water hole for at least 2 weeks (typically late March–early April). Camera traps were set up in the main areas of activity at the water hole and set to run 24 h/day, taking 60-s videos during daylight hours and 15-s clips at night, with a 1-s pause between successive videos.

### Behaviour coding

From these camera trap videos, as well as from additional handheld camcorder videos taken ad libitum during focal individual follows between 2011 and the present, we coded all digging-related behaviour (Table 1), as well as individual identity, age category, sex, and for peering at well-digging events, the identity of the observer and observed individual. Age categories were defined as follows: infant (0–4 years), juvenile (5–9 years), subadult (10–14 years for females, 10–15 years for males), and adult (>14 years for females, >15 years for males) following Reynolds (2005). We define well-digging as the manual scraping of substrate next to an open water source in order to dig a small hole, which then fills with water that the individual drinks either directly or with a drinking tool. The behaviour has two essential parts, digging and drinking, with an optional waiting period between the two depending on water levels and how fast the water seeps into the well.

**Table 1:** Definitions of the four possible digging-related events [play digging, digging, well-digging, and peering at well-digging (*Peering*)] coded from the videos

Behaviour	Definition
Play digging	Manually scraping the substrate or mud next to open water in an ineffective, playful manner–movements are variable and imprecise, and often involve the whole arm; fingers are held straight or relaxed
Digging	Manually scraping the substrate next to open water; small, controlled movements with slightly bent fingers. Even where a hole is produced, the individual does not drink from it
Well-digging	Manually scraping the substrate next to open water; small, controlled movements with slightly bent fingers. The individual drinks the water that filters into the resulting hole, either directly or with a drinking tool
Peering	Directly looking at another individual well-digging for at least 5 s at a close enough range that enables the peering individual to observe the action in detail; it is indicated by the orientation of the head of the observer when following the actions of the observed individual [in accordance with Schuppli et al. (2016)]

## Results

A total of 56 digging-related events were coded from 121 videos (Table 2); the average number of events per individual was 2.8 ± 3.3 (range = 1–15 events per individual). We defined an event as a bout of behaviour targeted at the well, with less than 1 min elapsing between consecutive videos, and without the individual leaving the frame. Seven events were excluded from the dataset because individuals were not fully identifiable due to poor or partial visibility. Twenty different individuals were identified: 13 females and seven males, of which eight were mature and 12 immature.

**Table 2:** Number of peering at well-digging, play digging, digging, and well-digging events recorded for each age-sex category

Age class	Sex	Peering at well-digging	Play digging	Digging	Well-digging
Adult	Female	2	0	1	22
	Male	0	0	0	0
Subadult	Female	0	0	2	2
	Male	0	0	0	1
Juvenile	Female	3	1	2	2
	Male	0	0	1	1
Infant	Female	5	1	1	0
	Male	2	4	3	0
Total		12	6	10	28

Age categories follow Reynolds (2005)

### Digging-related behaviours

**Fig. 2:**
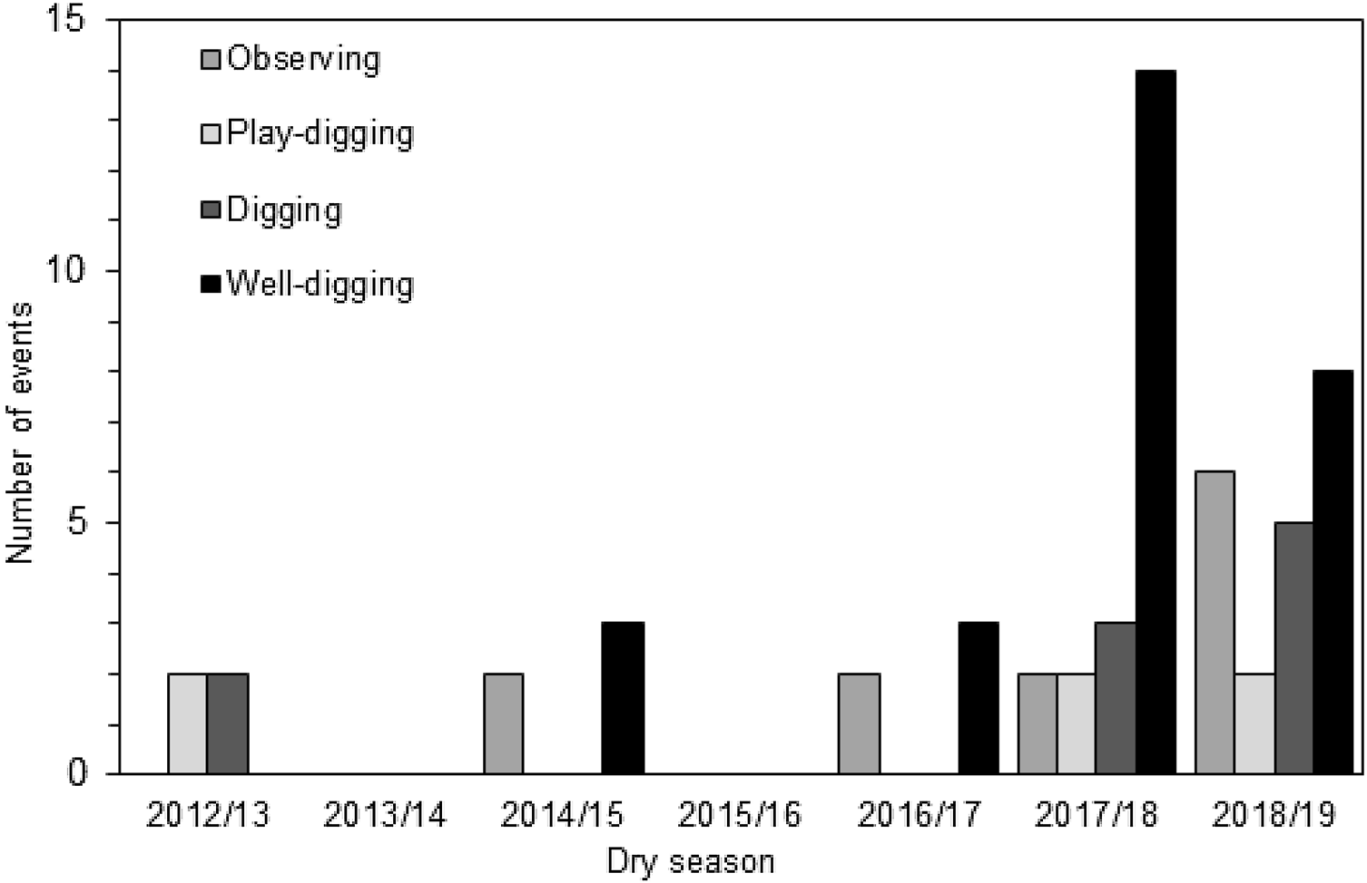
The number of recorded digging-related behaviours [peering at well-digging (*Observing*), play digging, digging, and well-digging] across the dry seasons 2012/2013 to 2018/2019. No well-digging was observed before 2014/2015; three well-digging events were recorded in 2016/2017, fourteen in 2017/2018, and eight in 2018/2019. Digging and play digging were recorded from 2012/2013; there were no recorded digging-related events of any kind in 2013/2014

Digging-related behaviours were recorded between 2013 and 2019, with play digging and digging reported in 2013 prior to ONY’s immigration. The first observation of well-digging occurred in February 2015 (Fig. 2). ONY showed no exploratory behaviour, but immediately dug a competent well (Video S1). She repeated the behaviour across that dry season and was the individual for which the highest number of well-digging events (*n* = 14) was recorded in the dataset. During the first direct observations of this behaviour by the field team in 2017, another parous adult female was recorded to observe ONY digging with apparent interest for at least 9 min [including peering (Video S2), note that the behaviour had already started prior to the first video], and wait for her to finish drinking. Over the next 4 h, two adult males, one adult female, and two immature males were observed to exploit the well with either a leaf sponge and/or for direct drinking (Video S3).

**Fig. 3:**
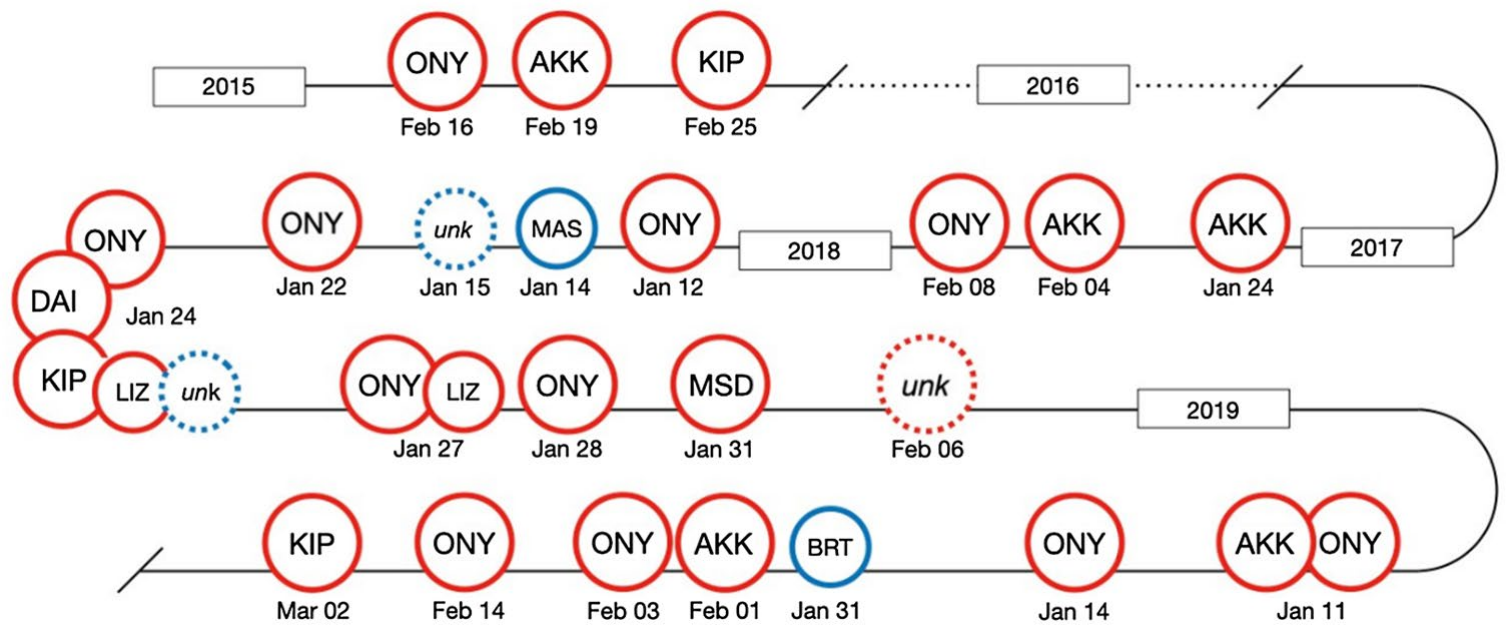
Timeline of recorded well-digging events across dry seasons 2012/2013 to 2018/2019, with the individual’s identity indicated.* Red circles* indicate females,* blue circles* indicate males,* large circles* indicate adult individuals,* small circles* indicate immature (subadult and juvenile) individuals.* Dotted circles* indicate individuals who could not be clearly identified [unknown (*unk*)]

The next individual observed well-digging was AKK, in 2015. Over the following 4 years, eight individuals were recorded well-digging, four of them repeatedly: two parous adult females, KIP (*n* = 3) and AKK (*n* = 5); the nulliparous adult female, ONY (*n* = 14); and a juvenile female, LIZ (*n* = 2), unrelated to any of the three adults (Fig. 3).

No tools were used to dig wells. Waibira chimpanzees employed a range of drinking techniques at the wells, including both direct drinking and drinking-tool use. Drinking tools included leaf sponges (newly made, or reuse of discarded sponges) and moss sponges, but no drinking tools were specific to drinking from wells. Leaves were also used to wipe mud from the hands after digging.

## Discussion

Chimpanzees living in water-restricted areas are able to exploit subsurface water by digging wells to access it (Nishida et al. 1999; McGrew et al. 2003; Hunt and McGrew 2002). Waibira chimpanzees were observed digging wells by hand next to a pool with stagnant surface water, their main water source during the dry season. We did not observe any use of tools for well-digging. Over a period of 377 days across seven annual dry seasons we documented habitual well-digging in four female chimpanzees. Importantly, this happened while stagnant surface water was available at the same time, suggesting they preferred the well water. Other individuals subsequently exploited the pre-dug wells for their own water access, either directly or by using sponges, again while (stagnant) surface water was available nearby.

Although digging behaviour (e.g. play digging) had been observed in the Waibira community prior to ONY’s immigration in 2015, we recorded no observations of well-digging in the community prior to 2015, despite camera trap video recording at the site (ongoing since January 2013) and direct observations during focal follows (ongoing since 2012). We also recorded no indirect evidence of well-digging at the water hole prior to 2015. While it is impossible to rule out that we missed this behaviour or failed to recognise the indirect traces of it, we consider it likely that ONY introduced the behaviour into the Waibira group. First, her competence and frequency of well-digging were remarkable from the beginning, suggesting that she knew the behaviour prior to immigration. Second, and equally remarkable, were the behavioural responses of other adult individuals who closely observed her well-digging behaviour and then exploited her wells over several years, suggesting that the behaviour was previously unknown to other Waibira adults.

A similar pattern was recorded in adult chimpanzees in Bossou, who closely observed (‘peering’) previously unknown nut-cracking behaviour in a field experiment (Biro et al. 2003). It has been argued that peering is a good indicator of ongoing social learning in apes (Schuppli et al. 2016), which is in line with our observations. Since its introduction in 2015, well-digging has now been observed repeatedly and in multiple individuals, suggesting it has spread–potentially by social learning–within the Waibira community. No similar behaviour has ever been observed in the well-studied neighbouring Sonso community (Reynolds 2005), despite three decades of careful observations and the fact that both groups occupy the same continuous forest habitat. Sonso chimpanzees, however, benefit from year-round access to a small river (the Sonso river), which flows across core areas of their territory and provides continuous access to fresh water.

Over the seven-season study period, we observed eight individuals to dig wells, but all four of the habitual well-diggers were females (three adults and one juvenile). While some younger males were observed to dig a well on at least one occasion, no adult male has so far been seen to dig one (although they were observed exploiting wells dug by others, suggesting a preference for the well water over the stagnant water that was still freely available). This female-biased pattern of spread is similar to that observed in Japanese macaques (*Macaca fuscata*) for potato washing (Nakamichi et al. 1998). Our camera trap coverage of the water hole is incomplete and it was not possible to observe all drinking events or social behaviours. Thus, it is likely that some well-digging and other digging-related behaviour were not captured in our video dataset. However, there is characteristic physical evidence of well-digging that is relatively easy to identify, such as the hole having clearly defined sides; the presence of separate marks made by fingers at the hole’s lip (where the fingers are initially dug into the soil) or in the area where the tailings remain; the presence of a small pile of substrate where the direction of digging with the fingers is consistent; in addition, the likelihood that well-digging has occurred is further supported by the presence of drinking tools in or around the hole (Video S4) (McGrew et al. 2007, 2013). During the same period of observation, the water hole and the surrounding area was surveyed regularly (at least once a week) for another study, and no indirect physical evidence of well-digging was observed prior to 2015. The failure to acquire this–very easily performed–behaviour by more individuals is puzzling, particularly since they have been observed to exploit the wells dug by other individuals. One possible explanation is that, while the physical act of well-digging for subsurface water is easy, the cognitive puzzle presented by its status as a concealed resource is more challenging, particularly given the (short) delay between the action of digging and the appearance of clean water, and the likely absence of clear cues to its presence. For example, in a muddy rainforest water hole olfactory cues to subsurface water presence are likely obscured by those from stagnant surface water. Delayed rewards or trace conditioning is shown to negatively impact the speed of learning compared to direct stimulus association (Kamin 1961; Beylin et al. 2001), and due to its significant cognitive demands, has been proposed as a possible test for animal consciousness (Shea and Heyes 2010)*.* Additional reinforcing factors, such as observing a conspecific well-digging, may facilitate recognition of the connection between the action of manual digging and acquiring clean subsurface water.

Given wider trends in the spread and maintenance of group-specific behaviour in chimpanzees, we predict that in the future we will see (1) further spread of well-digging between adult females and immature individuals of both sexes; (2) matrilineal spread, as three of the habitual well-diggers were mature females, two of them with offspring; and (3) the possible spread to adult males with the maturation and rise in rank of immature male well-diggers.

In conclusion, we describe a new case of well-digging in chimpanzees–the first described for a rainforest-living group. We describe the apparent spread of this behaviour, which was potentially introduced by an immigrant female. The repeated innovation of well-digging across four communities of chimpanzees (McGrew et al. 2003, 2007; Hunt and McGrew 2002) could be explained by individual learning in response to a strong ecological necessity; however, the apparent absence of this behaviour in Waibira prior to ONY’s immigration, followed by its subsequent rapid acquisition by a few–but not many–group members, suggests that there was a socially mediated component to its spread in Waibira. Our observations support previous evidence suggesting that social transmission typically occurs to other younger or low or similarly ranked individuals (Horner et al. 2010). The now habitual use of a technique previously associated with communities that live in savannah or savannah-woodland highlights the importance of seasonal variation as well as broad ecological variation in resource availability for forest-dwelling chimpanzees (Wessling et al. 2018). Irrespective of the typical availability of water as a resource, the limited presence of water during at least some periods of the year appears sufficient to shape chimpanzee behaviour in the Waibira community. Taken together, these observations highlight both the striking variation and flexibility of chimpanzee behavioural repertoires.

## Supplementary Information

Below is the link to the electronic supplementary material.

Supplementary file1 First observation (from camera trap) of ONY digging on 16 February 2015. First observation (from camera trap) of ONY digging and then drinking from a well at the water hole. While the large adult male on the right sits down, ONY—the third individual from the left—is visible at 1 s digging in the background. She digs until 11 s in, at which point she starts to drink and does so for the remainder of the video. The adult female LIR is sitting to the right of ONY and can be seen watching her throughout. (MP4 56600 KB)

Supplementary file2 First observation (direct) of ONY digging on 8 February 2017 at 11.40 a.m. ONY is seated on the left and is clearly seen digging from the start of the video. Another adult female (KET, with her infant KYO) is seen on the right, making, then using, a leaf sponge while facing ONY. (MP4 23514 KB)

Supplementary file3 Continuation of first observation (direct) of ONY digging on 8 February 2017 at 11.42 a.m. ONY is seen on the left picking out an object and then digging to enlarge the well, waiting for water to rise within it, and then drinking from it for several minutes. KET, who is dipping a leaf sponge into water and drinking from it, and her infant KYO, can be seen on the right. At 3 min  49 s, KET, who has been facing ONY and, together with her infant, KYO, watching her intermittently, then approaches to a near distance and watches her more intently (peering, e.g. from 3 min 56 s to 4 min 38 s), but does not use the well. Both females leave at the end of the video (7 min 23 s). (MP4 192231 KB)

Supplementary file4 LIR and offspring drinking and leaf sponging from the well dug by ONY on 8 February 2017. At 1.43 p.m., the adult female LIR is seen drinking from the well that ONY dug several hours earlier. LIR’s infant, LAM, is seen peering to the left of LIR. At 20 s, LAM puts leaves into his mouth as though to make to make a leaf sponge, but continues to chew on the leaves picked, and adds others to the wadge in his mouth (including those of non-food species; 54 s); he is not seen to use the leaves to sponge up water, and appears to have swallowed them by 1 min 20 s. At 1 min 2 s, LIR makes a leaf sponge by incorporating two types of leaves and then uses it in the well, and LAM peers at her while she does so. At 1 min 38 s, LIR’s juvenile son, LKU, moves into the frame and immediately drinks from the well. At 1 min 53 s, LAM dips his fingers into the well and drinks from them. (MP4 60421 KB)

Supplementary file5 Adult male MOR drinking from the well dug by ONY on 8 February 2017. At 2.11 p.m., the adult male MOR is seen drinking from the well dug by ONY several hours earlier. (MP4 19851 KB)

Supplementary file6 Adult male ILA drinking from the well dug by ONY on 8 February 2017. At 4.02 p.m., adult male ILA is seen drinking from the well dug by ONY several hours earlier. (MP4 6816 KB)

Supplementary file7 A well dug by adult female ONY on 12 January 2018 in the sand/gravel substrate of the water hole. The hand of CH (small adult human hand, wrist to tip of the middle finger, length 17.5 cm) is shown for scale. Discarded leaf sponges can be seen in and around the water hole. (MOV 230879 KB)

## Data Availability

All data are available in the manuscript.
